# A population-based study of breast cancer prevalence in Australia: predicting the future health care needs of women living with breast cancer

**DOI:** 10.1186/1471-2407-14-936

**Published:** 2014-12-11

**Authors:** Xue Qin Yu, Roberta De Angelis, Qingwei Luo, Clare Kahn, Nehmat Houssami, Dianne L O’Connell

**Affiliations:** Cancer Research Division, Cancer Council New South Wales, Sydney, Australia; Sydney School of Public Health, University of Sydney, Sydney, Australia; Centro Nazionale di Epidemiologia Sorveglianza e Promozione della Salute (CNESPS), Istituto Superiore di Sanità, Rome, Italy; School of Public Health and Community Medicine, University of NSW, Sydney, Australia; School of Medicine and Public Health, University of Newcastle, Newcastle, Australia

**Keywords:** Breast cancer, Cancer survivorship, Cancer prevalence, Incidence, Statistical projection, Epidemiology, Australia

## Abstract

**Background:**

Breast cancer places a heavy burden on the Australian healthcare system, but information about the actual number of women living with breast cancer and their current or future health service needs is limited. We used existing population-based data and innovative statistical methods to address this critical research question in a well-defined geographic region.

**Methods:**

Breast cancer data from the New South Wales (NSW) Central Cancer Registry and PIAMOD (**P**revalence and **I**ncidence **A**nalysis **MOD**el) software were used to project future breast cancer prevalence in NSW. Parametric models were fitted to incidence and survival data, and the modelled incidence and survival estimates were then used to estimate current and future prevalence. To estimate future healthcare requirements the projected prevalence was then divided into phases of care according to the different stages of the survivorship trajectory.

**Results:**

The number of women in NSW living with a breast cancer diagnosis had increased from 19,305 in 1990 to 48,754 in 2007. This number is projected to increase further to 68,620 by 2017. The majority of these breast cancer survivors will require continued monitoring (31,974) or will be long-term survivors (29,785). About 9% will require active treatment (either initial therapy, or treatment for subsequent metastases or second cancer) and 1% will need end of life care due to breast cancer.

**Conclusions:**

Extrapolating these projections to the national Australian population would equate to 209,200 women living with breast cancer in Australia in 2017, many of whom will require active treatment or post-treatment monitoring. Thus, careful planning and development of a healthcare system able to respond to this increased demand is required.

**Electronic supplementary material:**

The online version of this article (doi:10.1186/1471-2407-14-936) contains supplementary material, which is available to authorized users.

## Background

Breast cancer is currently the most common cancer among women worldwide [1], and is expected to remain so in the foreseeable future [2, 3]. In Australia, the risk of a woman developing breast cancer before the age of 85 is 1 in 8 [4], and the number of new diagnoses is expected to continue to increase in the future [5]. Fortunately however, advances in diagnosis and treatment mean that breast cancer survival is now very high [6]: the 5-year relative survival in Australian women was 89.4% in 2006–2010, and for those diagnosed with small tumours (the majority of the screen-detected tumours) 5-year relative survival was over 98% [4]. As a consequence of these trends of rising incidence and survival, it is almost certain that the number of Australian women living with breast cancer will keep increasing in the near future. Understanding the health-care needs of this growing population and the subsequent demands on health-care will enable better allocation of resources and the provision of better care, and is therefore of increasing importance.

Despite these predictions, there is currently only very limited information available about breast cancer prevalence and the current or future health service needs of breast cancer patients in Australia. Information available tends to be restricted to the number of prevalent cancer patients at a past date [7, 8], which is of limited use in predicting future health service requirements. Current and future estimates of prevalence would be more useful for health service planning, but as estimating cancer prevalence is a complex process, reliant on accurate incidence and survival modelling, this information is rarely available.

Predicting future breast cancer health service needs is further complicated by the widely varied treatment and follow-up requirements of these women [9, 10]. The population of survivors consists of individuals with varying needs: some may be in remission (needing follow-up care and surveillance), others may be receiving primary treatment after initial diagnosis, while others may be receiving treatment for metastases and some may be dying from breast cancer. Thus, estimates of cancer prevalence for relatively homogeneous populations of survivors defined by phase of the disease and who are likely to have similar healthcare needs would be informative for health service planning purposes. The aim of this study was to estimate the number of women living with breast cancer in Australia at different phases of the disease trajectory, and to predict their current and future health service needs.

## Methods

### Overview

There were three principal activities involved in this study: the estimation and projection of the prevalence of breast cancer, the analysis of phase of care prevalence, and the estimation of additional care needs for women with disease progression or second breast cancer. The data and methods involved in each of these activities will be described in detail below. In brief, to estimate and project complete prevalence of breast cancer we used the PIAMOD software (**P**revalence and **I**ncidence **A**nalysis **MOD**el) [11], with the primary input being first primary breast cancer incidence data for cases diagnosed in New South Wales (NSW) Australia. We then divided the estimated complete prevalence into four phases of care according to the different stages of the survivorship trajectory, and finally incidence data for subsequent metastases or second primary breast cancer were used to estimate the future prevalence of such events and the associated additional treatment requirements.

### Ethics statement

This study involves analysis of routinely collected data and the records were de-identified (name, address, date of birth had been removed) before being provided to the research team. The ethics committee waived the conditions for consent because it is impracticable to seek consent as a large proportion of the individuals would likely have moved or died since their diagnosis of cancer which could be up to 40 years ago. Ethics approval was obtained from the NSW Population and Health Service Research Ethics Committee (reference number: 2009/03/139).

### Estimation and projection of prevalence

The PIAMOD software was used to estimate the observed prevalence (1972–2007) and project future prevalence (2008–2017). The PIAMOD method, described in detail by Verdecchia et al. [11], estimates and projects cancer prevalence and mortality through transition rate equations that relate prevalence and mortality to incidence and relative survival functions. It has been used to estimate and project cancer prevalence for many populations [3, 12–16]. The input files required by PIAMOD are population data, all-cause mortality, cancer-specific incidence and model-based survival estimates.

Incidence data for first primary female breast cancer (ICD-O3 C50) [17] diagnosed in 1972–2007 were extracted from the NSW Central Cancer Registry database. We included cases aged 18–84 years at diagnosis, and excluded cases who were reported to the registry through death certificate only, or who were first identified post-mortem. All-cause mortality data for NSW by single year of age (up to 84 years old), and calendar year (1972–2007), and corresponding mid-year NSW residential female population data by single year of age and calendar year were obtained from the Australian Bureau of Statistics.

### Modelling incidence data

Age, period and cohort (APC) models were fitted to the incidence data using a log-linear regression model implemented in the PIAMOD software. Nine relatively simple models (APC101, 102, 201, 202, 103, 301, 203, 302 and 303) were fitted and the most appropriate model was selected based on the likelihood ratio statistic (LRS) combined with knowledge of the epidemiology of breast cancer in Australia. The parameters of the chosen APC model were estimated using observed incidence for 1972–2007 and then this model was used for forward (after 2007) and backward (before 1972) projections. The resulting fitted incidence estimates were used as inputs for estimating future prevalence (for 2008–2017).

### Modelling survival data

Incident cases were followed up for survival status to 31 December 2007 (the most recent data available to us) through record linkage of the cancer cases in the Cancer Registry with the death records from the NSW Register of Births, Deaths and Marriages and the National Death Index. A two-step procedure was used to model the survival data. First, relative survival was estimated and tabulated, and then a mixture cure model was fitted to the tabulated relative survival estimates. Relative survival was tabulated using the Pohar Perme actuarial estimator [18], with the classic cohort approach for five calendar periods of diagnosis (1972–1980, 1981–1989, 1990–1995, 1996–2001, 2002–2007) and three age groups (18–49, 50–69, and 70–84 years). A mixture cure model was fitted to these tabulated survival data [19], and the survival estimates obtained from the model were then projected backward assuming a constant trend before 1972 and extrapolated forwards for 2008–2017 assuming that cancer survival trends will continue as previously observed. The model-based estimates of survival from the mixture cure model were used as inputs into PIAMOD for the next step of the analysis.

### Prevalence estimation

Using the PIAMOD software and the prepared input data for the estimated incidence and survival, as well as all-cause mortality and population data we were then able to calculate the prevalence of first primary breast cancer for 1972–2007 and to estimate the future prevalence for 2008–2017. Because PIAMOD can only provide results for closed age groups and populations, and as the available data for the older population were grouped for those aged 85 years and over, our prevalence estimates include cases up to age 84 years only. Population projections after 2007 were derived in PIAMOD by assuming birth rate and mortality for causes other than the specific cancer to be stable over time [11].

### Validation of PIAMOD estimates

A validation of the overall estimation procedure was performed using external data that were not used in the modelling. In this case we compared the expected breast cancer mortality derived by PIAMOD with the observed mortality in NSW. This offers an overall validation of both the incidence APC model and of the relative survival function. Good agreement between the expected mortality and the observed mortality means that the relative survival function correctly modulates the relationship between incidence and mortality.

### Phase of care analysis

**Figure 1:**
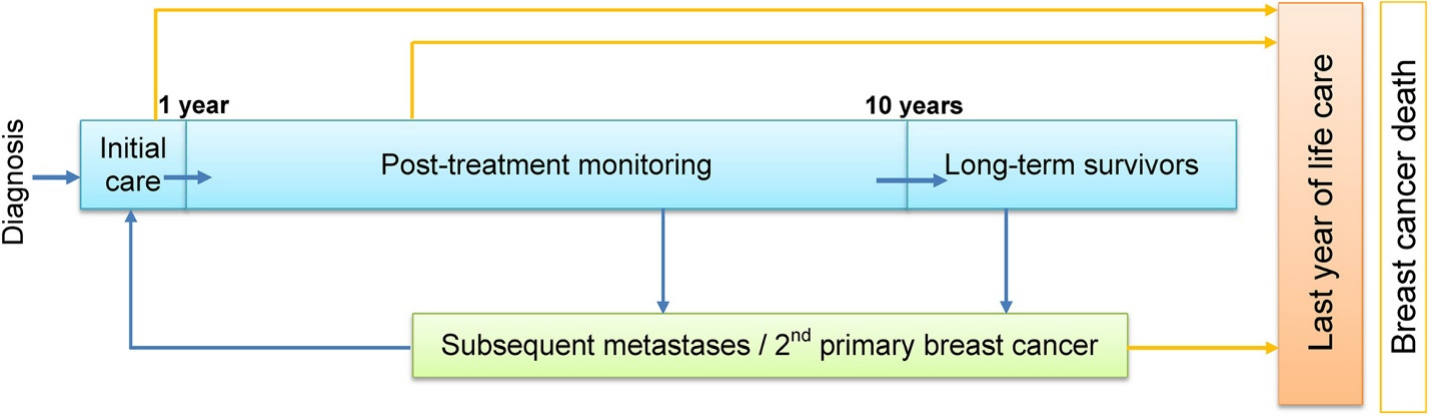
**Pathways of the breast cancer survivorship journey.**

The estimated complete prevalence was decomposed into four primary phases of care according to time since diagnosis, year of death and cause of death. These phases of care were the initial care phase, the post-treatment monitoring phase, long-term survivors and the last year of life phase, as illustrated in Figure 1.

The *initial care phase* was defined as care provided in the first 12 months after diagnosis (excluding cases who died within the first year after diagnosis). The *post-treatment monitoring phase* was defined as the period after initial care and before being considered a long-term survivor.

The definition of long-term survivors varies in the literature and across cancer types. Long-term survivors are often considered to be cancer patients who have lived beyond 5 years after diagnosis [20–22], but the patterns of breast cancer survival and recurrence indicate that a longer time since diagnosis may be more appropriate for defining long term survival of breast cancer. Thus, similar to other researchers [23], we defined *long-term survivors* as those who survived at least 10 years after diagnosis.

The *last year of life phase* was defined as the last 12 months of life for those who died of breast cancer. Cases with short survival (less than 12 months) were considered to be in the last year of life phase. We used information on cause of death to identify those patients who had died from breast cancer in a given year, and who would therefore be in the *last year of life* phase of care in that year. Future numbers of cases in the *last year of life phase* for 2008–2017 were determined by the projected breast cancer mortality trend (derived from PIAMOD method).

In addition to these four primary phases of care, an additional sub-phase of care (*treatment for metastases/second cancer*) was created to account for cases in the post-treatment monitoring and long-term survivor phases who require more treatment at some point during follow-up due to tumour spread or the development of a second breast cancer.

### Estimation and projection of metastases or second primary breast cancer

Cases diagnosed with first primary breast cancer in 1972–2007 were followed up for subsequent metastatic spread or second breast cancer to the end of 2007. The development of metastases was identified using subsequent notifications from 120 days after the first diagnosis. As it is challenging to identify and accurately distinguish between subsequent metastases and second primaries using population datasets, and it is likely that all such cases will require further treatment, we combined the counts of second breast cancer and metastatic tumours.

To estimate the number of these events in the future, we first calculated the proportion of cases in the post-treatment monitoring and long-term survivor phases who presented with subsequent metastasis or second breast cancer in 2006. We then applied this proportion to the number of projected cases in the post-treatment monitoring and long-term survivor phases in 2008–2017. Those patients who survived at least one year after the diagnosis of subsequent metastases or new primary breast cancer were categorised into the treatment for metastases/second cancer phase. Those who died within one year after the diagnosis of a metastases or new primary breast cancer were considered to be in the last year of life phase.

While each patient can contribute to more than one phase of care over time, at any one specific point in time a patient can only be in one phase of care in the analysis.

## Results

### Incidence trends

**Figure 2:**
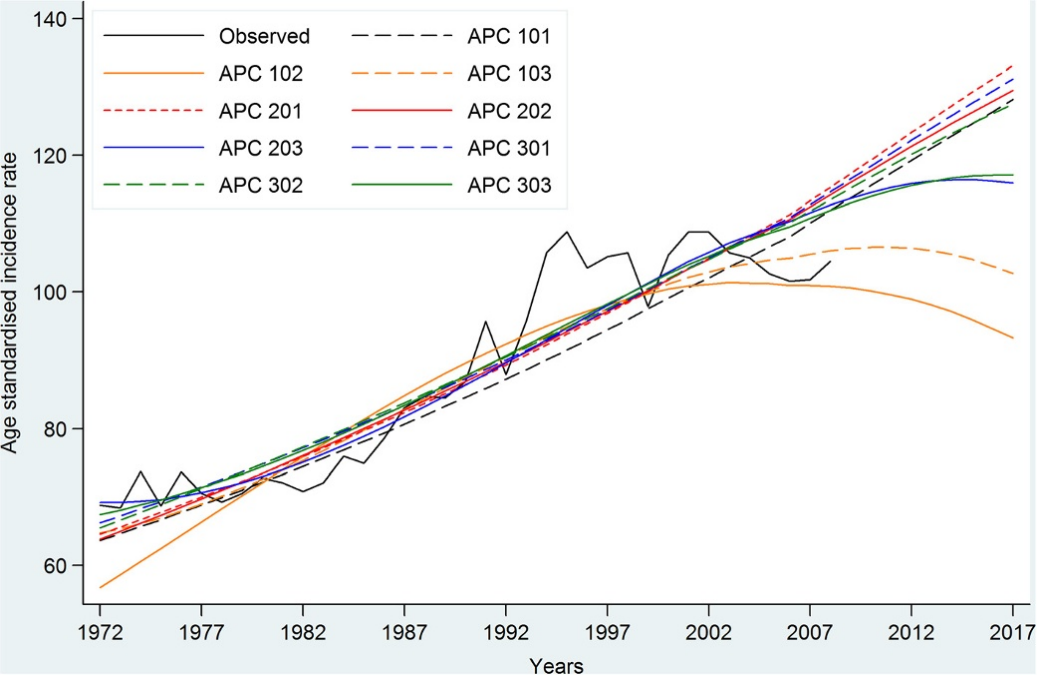
**Comparison of Age-Period-Cohort incidence models and observed age-standardised incidence rates for breast cancer in NSW Australia.**

**Figure 3:**
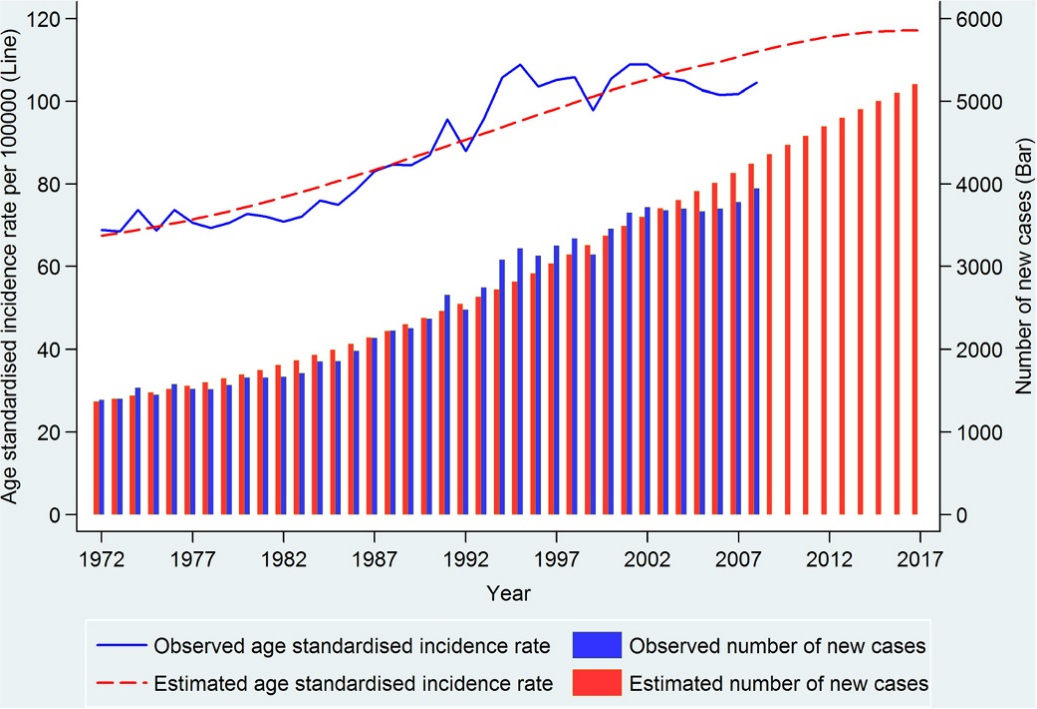
**Observed breast cancer incidence in NSW Australia for 1972–2007, and projected incidence for 2008–2017.**

A total of 89,768 cases of first primary breast cancer diagnosed in 1972–2007 were included in the incidence and prevalence analyses. The observed incidence trend can be summarised with four different patterns: a relatively stable period (1972–1985), a moderate increase (1986–1992), a more rapid increase (1993–1995), and then stabilisation at a high level after 1996. During the more stable period from 1996 there are a few fluctuations in incidence, likely due to random variation and the reduction in hormone-replacement therapy use that occurred in Australia [24, 25], and in many other developed countries [26], after the publication in 2002 of the results of the Women’s Health Initiative randomised trial [27]. The increased incidence between 1985 and 1996 was most likely the result of mammographic screening, with informal screening occurring between 1985 and 1992 [28] and a population-based screening program introduced in NSW from 1992 [29] (Figure 2). We plotted the estimated incidence from nine APC models against the observed incidence (Figure 2). Based on national breast cancer projections [4] and more recent NSW data [30], the APC model 303 (age^3^ and cohort^3^) was considered to be the most appropriate model with which to project incidence for 2008–2017. This was supported by model 303’s much smaller LRS value than those of APC models 203 and 302 (Additional file 1), which indicates that it is a better fitting model. Thus, estimated and projected incidence from this model (shown in Figure 3) were used as inputs for the projection of prevalence.

### Survival trends

**Figure 4:**
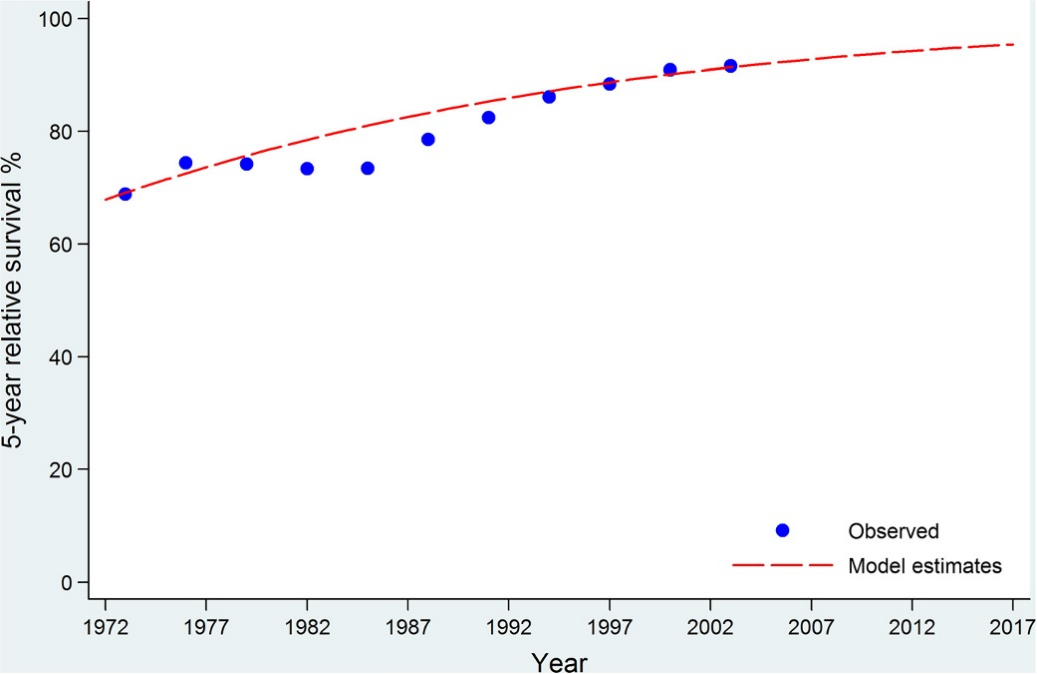
**Comparison of fitted five-year breast cancer relative survival with observed for 1972–2007 and projected survival for 2008–2017 in NSW Australia.**

Observed and fitted five-year breast cancer relative survival trends over time (assuming a constant trend before 1972 and dynamic trend after 2007) are shown in Figure 4. It can be seen that survival improved markedly from 1985 to 1997, followed by a slower increasing trend after 1997.

### Validation of PIAMOD incidence and survival estimates

**Figure 5:**
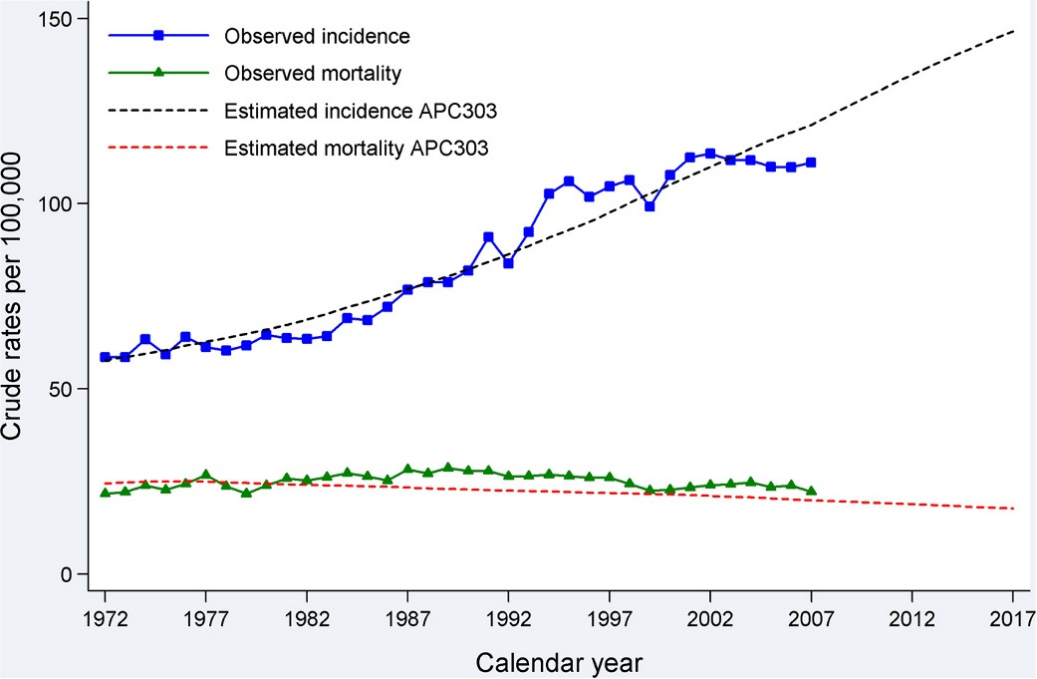
**Comparison of fitted crude breast cancer incidence and mortality with observed crude incidence and mortality for 1972–2007 and projected incidence and mortality for 2008–2017 in NSW Australia.**

Validation of the chosen APC incidence model and the modelled relative survival estimates (Figure 5) indicates that the APC model fitted the observed incidence data well, which is supported by the reasonably good agreement of the expected mortality with the observed mortality.

### Projected prevalence

Since 1990, the number of breast cancer survivors aged 18–84 years in NSW has increased over 150%; from 19,305 in 1990, to 35,538 in 2000, and then to 48,754 in 2007. This number is projected to increase further to reach 68,620 in 2017, with an annual rate of increase of 4.07% (Table 1) from 2007 to 2017. The expected increase in the number of prevalent cases was greatest for the oldest age group, with a 61.7% increase from 2007 to 2017. Those aged 50–69 years showed an expected 40.9% increase. The effect of population ageing can also be seen in Table 1: the youngest age group made up about 13% of the total prevalent cases in 2007, but this proportion is expected to decrease to 8% by 2017, while the proportion of prevalent cases aged 50–69 years is expected to remain unchanged over the same period.

**Table 1:** **Age and year-specific estimates of prevalence of breast cancer in NSW Australia**

Year	Number (%) of woman living with breast cancer						
	<50 years		50-69 years		70-84 years		Total
2007	6204	(12.7%)	26,265	(53.9%)	16,285	(33.4%)	**48,754**
2011	5774	(10.5%)	29,881	(54.3%)	19,373	(35.2%)	**55,028**
2013	5609	(9.4%)	32,606	(54.9%)	21,185	(35.7%)	**59,400**
2015	5420	(8.5%)	35,062	(54.9%)	23,429	(36.7%)	**63,912**
2017	5292	(7.7%)	37,003	(53.9%)	26,325	(38.4%)	**68,620**

Estimates of phase of care prevalence in 2017 are presented in Table 2, and show that the majority of breast cancer survivors in 2017 will require post-treatment monitoring (31,974) or will be long-term survivors (29,785) who will need relatively less intensive follow-up. Age-specific estimates indicate that the majority of the cohort (54%) will be those aged 50–69 years and the largest single group will be those under post-treatment monitoring aged 50–69 years, representing 28% of the total cohort in 2017.

**Table 2:** **Estimated numbers of women living with breast cancer in NSW Australia in 2017 by phases of care and age group**

Phase of care	Number (%) of breast cancer survivors							
	<50 years		50-69 years		70-84 years		Total	
Initial care	788	(14.9%)	2881	(7.8%)	1296	(4.9%)	4965	(7.2%)
Post-treatment monitoring	3531	(66.7%)	19,171	(51.8%)	9272	(35.2%)	31,974	(46.6%)
Long-term survivor	807	(15.2%)	14,031	(37.9%)	14,947	(56.8%)	29,785	(43.4%)
Last year of life care	77	(1.5%)	336	(0.9%)	361	(1.4%)	774	(1.1%)
Subsequent metastases /second tumour	89	(1.7%)	584	(1.6%)	449	(1.7%)	1122	(1.6%)
Total	5292	(8.0%)	37,003	(54%)	26,325	(38%)	68,620	(100%)

### Care for subsequent metastases or second breast cancer

Among the 89,768 women diagnosed with first primary breast cancer between 1972 and 2007 in NSW, there were 13,585 women (15.1%) who developed metastatic disease by the end of 2007. In addition, 9390 women had a second primary breast cancer. After excluding those who died within 12 months of the diagnosis of either second primary or metastatic disease, 491 (2.1%) women in post-treatment monitoring and 292 (1.5%) long-term survivors in 2006 would require additional treatment for their metastases or second primaries. Thus, by applying these two estimated proportions to the numbers of projected cases in the post-monitoring and long-term survivor phases in 2017, it is estimated that 1122 women would need further treatment due to their metastases/second primaries in 2017 (Table 2).

## Discussion

By estimating current breast cancer prevalence and providing projections of this prevalence in the future, this study fills a gap in Australian cancer research and provides a broad measure of the future health care needs of women with breast cancer in Australia. Our projected trends in prevalence indicate that the number of women living with breast cancer in NSW will increase by more than 40% from 2007 to 2017. Extrapolating these projections to the national Australian population would equate to 209,200 women living with a previous breast cancer diagnosis in 2017, many of whom will require treatment or post-treatment monitoring and related care [9, 10]. This information is useful for health policy makers and health service planners, ensuring that planning for future cancer care requirements is guided by appropriate evidence. It is also relevant to clinicians who provide care to breast cancer survivors throughout these phases, and may be of interest to the increasing population of breast cancer survivors.

Our prevalence estimate for 2007 in NSW using the direct counting method (1435 per 100,000) was consistent with the most updated national breast cancer prevalence estimate (1416 per 100,000) in 2007 reported by the Australian Institute of Health and Welfare (AIHW) [7]. The small difference between these estimates is likely to be because the AIHW used 26-year prevalence while ours was an estimate of 36-year prevalence. The similar definition of prevalence used in our study and the AIHW report (persons with multiple cancers being only counted once in the calculation) and the overall consistency of our prevalence estimate for 2007 (the most recent data available to us) with the most updated national breast cancer prevalence [7] provides indirect confirmation of our estimate. However, our study extended these results by using a valid statistical model (PIAMOD) to project future prevalence, which is more useful for health service planning for cancer patients.

Studies of breast cancer prevalence in the United States (USA) using SEER data have reported projected increases in prevalence comparable to our results, with the number of women with breast cancer in the USA expected to increase by an annual rate of 3.11% from 2010 to 2020 [3]. It is not surprising that these results are similar, as the main factor in breast cancer prevalence modelling is the incidence rate, and in both our study and the USA study incidence rates were predicted to remain at the current high level in the foreseeable future [31]. Also, the population age structures of the USA and Australia are broadly similar [32], and this is another important contributor to prevalence estimates.

This study is unique in its inclusion of data on subsequent cancer spread and second breast cancers to allow for the projection of prevalence according to phase of care. These two groups of patients with distant metastases or a new primary breast cancer constitute over 1100 women who will require active cancer treatment in 2017 in NSW, so it is essential that they be included when estimating future prevalence to inform cancer care needs. Furthermore, it is possible that due to the issue of incomplete episode data the reported number of patients with subsequent metastatic disease is an under estimate of the true figure [15, 33]. Data on cancer spread after initial diagnosis are not routinely collected by population-based cancer registries worldwide, but where possible the use of such data in research is a useful step towards providing clinically relevant information for patients, clinicians and health policy makers. Our results also provide some support for the ongoing surveillance of breast cancer survivors given the observed numbers with subsequent metastases from the first cancer and the emergence of second breast cancers, although also noting that these represented a modest proportion of women living with breast cancer. Oncologists and clinical researchers may be interested in our projected increased proportion of older breast cancer survivors, a group typically not included in clinical trials, and might consider expanding age criteria for current and future clinical trials.

Cancer prevalence is a function of cancer incidence and survival. As indicated by our model, the number of new breast cancer diagnoses will keep rising in the future (although the rates had started to stabilise), and survival is likely to continue to show some improvement, meaning that prevalence will inevitably also increase in the future. Our assumptions of future incidence and survival trends appear to be reasonable as they were based on 36 years of data and our understanding of the epidemiology of breast cancer in Australia. Our validation using external mortality data suggests that our projections for incidence and survival are likely to be appropriate (Figure 5). Most international studies, including those from the UK, Europe and the USA, indicated an increase in breast cancer prevalence in the future [2, 3, 34]. Therefore, these projections are likely to be relatively reliable, although as with all statistical predictions some uncertainties will remain.

While we have attempted to provide a robust estimation of breast cancer prevalence by phase of care we are aware that there are several limitations to this study. First, the PIAMOD software does not provide measures of uncertainty for projections of relative survival, population size and mortality, so we cannot assess the potential range of results. Second, not all changes in trends of cancer incidence and survival can be fully captured by our models, particularly for survival data (that even 10 years after diagnosis the probability of survival does not reach that of the general population) [35, 36]. However, different assumptions of future survival trends only had a small impact on the predicted prevalence (data not shown) because survival has less room for further improvement (five-year relative survival being over 90%). Third, we are aware that our projections are likely to underestimate future prevalence because we did not include cases aged 85 years or over (approximately 4% of the total patient population). We were unable to include these older cases because the PIAMOD software can only provide estimates for 1-year age groups, while the population was grouped as 85 years and over. Finally, although the phases of care definition used here is useful to infer future health care needs, the phases are often not as discrete as the categories imply, and some of them are cross-cutting, so that there are actually many different possible pathways cancer patients may experience from diagnosis to survival or end of life.

## Conclusions

As the Australian population ages the number of women living with breast cancer will increase, and consequently demands on health care services will also increase. In order to ensure adequate access to quality care for all future patients, careful planning and development of a healthcare system able to respond to this increased demand is required. Such preparation is critical, especially as the consequences of not providing appropriate cancer care and follow-up are already becoming apparent [37], and indeed, any shortfall in the oncology workforce could threaten the quality of patient care and safety [38]. In addition, a major investment in the infrastructure required to deliver cancer care is needed [39], and the rapidly increasing cost of cancer care must also be considered. A 27% increase in the national cost of cancer care was projected from 2010 to 2020 in the USA, with the largest increases being for female breast cancer and prostate cancer [3]. Australia must begin to consider how it will afford to provide quality cancer care for a large and increasing cancer survivor population in the future.

## Electronic supplementary material

Additional file 1: **Appendix Evaluation of the model-fit for age-period-cohort models for breast cancer incidence in NSW Australia 1972-2007**. (PDF 31 KB)

## Abbreviations

PIAMOD: Prevalence and Incidence Analysis MODel
NSW: New South Wales
APC: age-period-cohort
LRS: likelihood ratio statistic
SEER: Surveillance, Epidemiology, and End Results program
AIHW: Australian Institute of Health and Welfare.
