# Supplementary material for: A population-based study of breast cancer prevalence in Australia: predicting the future health care needs of women living with breast cancer
Source: BMC Cancer. 2014 Dec 11;14:936. doi: 10.1186/1471-2407-14-936 (PMC4295409; doi:10.1186/1471-2407-14-936)
Supplement: Supplementary file 1 — Additional file 1: Appendix Evaluation of the model-fit for age-period-cohort models for breast cancer incidence in NSW Australia 1972-2007. (PDF 31 KB) [file 12885_2014_5097_MOESM1_ESM.pdf]

Appendix Evaluation of the model-fit for age-period-cohort models for breast cancer incidence in NSW Australia 1972-2007

APC MODEL[1]	Number of parameters	Likelihood ratio statistic
303	7	3165
302	6	3450
301	5	3467
203	6	4333
202	5	5362
201	4	5379
103	5	12685
102	4	15666
101	3	22838

[1] X0Y denotes a polynomial model with age to the power x and cohort to the power y;
